# A new intervention for people with borderline personality disorder who are also parents: a pilot study of clinician acceptability

**DOI:** 10.1186/s40479-016-0044-2

**Published:** 2016-09-08

**Authors:** Kye L. McCarthy, Kate L. Lewis, Marianne E. Bourke, Brin F. S. Grenyer

**Affiliations:** Illawarra Health and Medical Research Institute, School of Psychology, University of Wollongong, Wollongong, New South Wales Australia

**Keywords:** Borderline personality disorder, Parenting, Brief intervention, Attitudes

## Abstract

**Background:**

Engaging parents who have a personality disorder in interventions designed to protect children from the extremes of the disorder supports both parenting skills and healthy child development. In line with evidence-based guidelines, a ‘Parenting with Personality Disorder’ brief intervention was developed, focusing on child safety, effective communication and parenting strategies.

**Method:**

Ratings of acceptability for the brief intervention model were given by 168 mental health clinicians who attended training. Changes in clinician attitudes, knowledge and skills were also assessed following training.

**Results:**

Providing clinicians treating personality disorder clients with additional skills to address parenting was well received and filled a gap in service provision. Clinicians reported improvements in clinical skills, knowledge, willingness and confidence to intervene in parenting issues with clients. Qualitative responses endorsed three major modes of learning: case study analysis, reflective learning activities, and skills-based intervention practices.

**Conclusions:**

Current treatment guidelines emphasise addressing parenting, but no evidence-based therapy includes specific parenting skills. This brief intervention model improved skills, efficacy and willingness to intervene. This approach can be readily added to current evidence-based therapy protocols and promises to improve client functioning and protect children from the extremes of the disorder. Clinical trials are now required to validate the approach in the field.

## Background

Parents with a mental illness deserve support, particularly those struggling with personality disorder who can also suffer considerable stigma. There are various effective psychological treatments for personality disorder, particularly Borderline Personality Disorder (BPD) [1], however none currently include a focus on parenting. The prevalence of parental mental illness has been estimated to be 21–23 % [2], whilst the risk for children of parents with mental illness is exacerbated to 41–77 % [3]. Significant levels of burden may be placed on children who take on the role of caring for a parent [4]. Thus, it is crucial that mental health providers are aware of their client’s status as a parent, and to assess and support their parenting capacity [2, 5–7].

Clinical guidelines for personality disorder include an emphasis on supporting parenting capacity [8]. For example, a recent clinical practice guideline for the management of BPD [9] states “People with Borderline Personality Disorder who have infants or young children should be provided with interventions designed to support parenting skills and attachment relationships” (recommendation 62), and further, “having Borderline Personality Disorder does not mean a person cannot be a good parent” (p.3). The core features of personality disorders and the onset of personality disorders from adolescence to early adulthood, and persistence throughout childbearing years [10], mean that maladaptive interpersonal patterns are likely to impact the parent-child relationship [11].

Despite this, protective factors for children and protective parental attributes have been identified within the literature [12]. The concept of ‘good enough’ parenting, that is, adequately responding to the child’s needs, has a long history in psychological literature, and supporting parents to do a ‘good enough’ job is particularly pertinent for parents with mental health disorders [13, 14]. While intervention programs for parents and caregivers with mood, anxiety, and substance abuse disorders have been shown to be helpful [15], the applicability of these programs to individuals with personality disorder is unknown. Attachment-based interventions may not address the parenting skill needs or goals of individuals with personality disorder [16]. Although numerous authors have made recommendations for the nature of interventions for parents with personality disorder (see [16–20] for review), interventions have yet to be developed.

### Description of the project air parenting intervention

To develop such a program was the impetus for this Project Air Strategy Parenting Project, where a wide range of existing parenting interventions were consulted in the development. This ‘Parenting with Personality Disorder’ brief intervention model provided a manual, associated resources and training for public mental health service clinical staff to assist people with BPD to strengthen and enhance their parenting skills. While primary treatment of the personality disorder is the main goal of any treatment, the interaction between mental health and parenting capacity underscores the need to jointly address these domains. Although the obvious focus is on BPD, the manual and approach is broadly cast as an intervention for people with a personality disorder, since people with other disorders (such as obsessive-compulsive personality disorder) are also likely to experience parenting difficulties where the intervention strategies presented here would be of benefit. It is likely that addressing parenting difficulties within the context of the mental illness will also improve mental health functioning, reduce family stress, and increase perception of parenting competence and fulfilment. This not only improves outcomes for parents with personality disorder, but is an essential component in providing care and protection for children and young people, which can contribute to the prevention of intergenerational mental health issues [21].

Several key principles formed the basis for the development of the Project Air Strategy Parenting Project brief intervention model (see Table 1) [22, 23]. The three strategies of the Parenting with Personality Disorder brief intervention model are outlined in Table 2, focused on supporting and developing safe relationships between parents and children by helping parents to increase their consistency in responding to children’s needs, and in a more reflective and sensitive manner. Given the diversity of professions and occupations of those working with people who have personality disorders, the brief intervention model was designed to be suitable for implementation by a range of clinicians including those with little expertise in working with people who have personality disorders, as well as those with greater expertise. The brief intervention is structured around the three strategy topics and the duration of the therapy is tailored to the individual needs of the individual. The shortest duration is three sessions (one session per strategy), but can be extended for up to four months to strengthen specific skills. The model has been designed to complement existing evidence-based therapies for BPD and can be easily inserted into existing programs.

**Table 1:** Project Air Strategy Key Principles for Clinicians Working with Parents with Personality Disorders

Prioritise child safety and encourage parents to do the same	
Listen to parenting struggles in a non-judgemental and accepting manner	
Focus on building trust and rapport, as parents with mental illness can feel vulnerable	
Recognise and value parents’ strengths and positive attributes	
Re-affirm that the goal is to be a ‘good enough’ parent, not perfect	
Help the parent to keep their child’s needs and feelings in mind despite mental illnesssometimes getting in the way	
Help parents to facilitate open discussion with their child about what is happening in the home, including discussing the parent’s mental health issues and their diagnosis	
Ensure a family crisis plan is in place for when the parent is very unwell	
Help parents with parenting skills, including age-appropriate ways of relating to their child and setting firm and kind limits to protect everyone	
Where possible seek opportunities to protect children from being distressed by mental illness	
Ensure children have the best possible chance to grow up normally, and prioritise ensuring they attend school and have time to join in with their peers

**Table 2:** Key Strategies of the Parenting with Personality Disorder Intervention

Strategy 1: Engage the parent to reinforce safety for all. Building a collaborative relationship regarding parenting; delivering key parenting messages; and completing a Family Crisis Care Plan to support child protection and family safety.	
Strategy 2: Improve communication and strive to separate parenting from personality disorder. Skills in talking to children about personality disorder, protecting children from personality disorder symptoms, setting firm but fair limits to reinforce safety and security.	
Strategy 3: Improve relationships. Reflect on the relationship between the parent and child, skills for mindful parenting and understanding emotions, reinforce the importance of treatment for mental health issues and self-care and self-compassion in parenting.	

The intervention is fully documented in a manual [23]. The aim of the program is to create the opportunity to reflect on parenting issues with clients with personality disorder and intervene to improve parenting. The intervention offers a multifaceted approach so that the parenting issues targeted in the intervention can vary, and the intensity of the intervention can be adjusted for individual parents, based upon the family’s need, the parents’ willingness to engage and the service’s capacity. For each step of the program, tailored fact sheets, care plans and treatment handouts are provided to reinforce key points and encourage deeper discussion.

The first strategy is to engage the parent to reinforce safety. Thus the first step in the intervention is to create motivation to participate by explaining how discussing parenting can assist in treatment, and building a collaborative understanding that addresses any fears or reluctance and ensures the family is protected. Often people with personality disorder can be anxious to discuss parenting because of self-criticism (being a ‘bad’ parent), feelings of inadequacy or even fears that their children may be removed from their care. Yet at the same time parenting can create additional stresses that make treatment more difficult. Early in the intervention parenting goals are elicited as a way to begin exploring both the parent’s strengths and their current challenges in meeting their children’s needs. A Family Crisis Care Plan is developed to put in place an agreement that ensures trusted adults are identified who can support the adequate care of children, should the parent become temporarily unwell or need a stay in hospital.

The second strategy is to improve communication and strive to separate parenting from personality disorder. Thus the second step in the intervention is to teach the parent how to talk to their child about their mental illness in an age-appropriate way. It is important that children understand their parent has a mental illness and they are not to blame for the illness. A third step is to develop strategies that protect children from the personality disorder symptoms. It can be distressing for children to witness a parent expressing powerful and overwhelming emotions. Similarly, being exposed to behaviours used by parents to cope can also be highly distressing and traumatic for children, including the consumption of drugs and alcohol, self-harm and suicidal thoughts or intentions, domestic violence and impulsive sexual behaviour. The focus is on working on strategies for how parents can keep some focus on their children’s safety in difficult moments, and avoid exposing children to these distressing behaviours. Strategies can include taking short breaks, considering day care or school assistance, and ensuring emotion support is obtained from other adults and services rather than from the child.

A fourth step in the intervention involves encouraging parenting that sets firm but fair limits with children to reinforce safety and security. Children respond well to simple and predictable daily routines so setting expectations about behaviour can reinforce a child’s security. Here it is important to help the parent find ways to let their child know that they are safe in the parent’s care, and that the parent is willing and able to take the parenting role. Similarly, it is important to help parents identify and step in when children take on too much responsibility. During this stage the therapist can help the parent to begin to talk about and consider children’s needs, and find ways for the parent to let their child know that they want them to act their age and do the things that interest them, including school, spending time with friends and hobbies.

A third strategy is to improve relationships. Thus a fifth step in the intervention involves reflecting on relationship patterns between parent and child and discussing the parent-child attachment bond and how this can be strengthened. It may sometimes be difficult for parents with personality disorder to consider the perspective of their child, or how their behaviour may be impacting on their child, and they may need encouragement, support or assistance to do this. Mindful parenting and encouraging positive play-time with the child can be effective strategies. Similarly, it can be helpful to consider discussing the ways that emotional communication can be confused or misunderstood between the parent and child, particularly when difficult or traumatic feelings from the parent’s past are triggered in the present.

A DVD audiovisual family case study is included in the program to facilitate reflection and practice of skills with clients (Table 3). As shown in Table 3 the case study allows discussion to be opened around the program strategies and intervention steps. The first scene allows discussion of separating parenting from mental health concerns. The second scene illustrates how mindful playtime can interrupt situations when the parent becomes overwhelmed by their memories and their emotional dysregulation spills over onto their children. The third scene opens the discussion about ensuring children are allowed to be children, reinforcing school attendance, and reducing the risk of children becoming parentified or carers of their parent.

**Table 3:** Family Case Study: Parenting with Personality Disorder

Sam is a 31 year old single mother of three children, Ethan aged 14 years, Jack aged 8 years, and Mia aged 3 years. Sam loves her children, and wants the best for them. Sam has been diagnosed with Borderline Personality Disorder, and has difficulties managing relationships and stormy emotions, often using self-harm to cope when she feels overwhelmed. Sam has been working with a mental health clinician to reflect on her relationships with her children and on the choices she makes in caring for them on a daily basis, as small actions can make a big difference in her family. Sam and her clinician have been talking about the following choices Sam has recently been faced with:	
1. Sam was in the bathroom and had cut herself. Jack was worried about where his mum was, and knocked on the door, asking her to let him in. Sam was feeling overwhelmed, alone, and was wishing for someone to support her, and her initial response was to open the door to seek support from Jack. However, she then took some time to reflect and consider her choices and how they would impact on her son. She then chose to tell Jack that she was ok and was washing her face, and would be out shortly. She asked him to watch TV with his sister. Sam engaged in some self-care, and washed and bandaged her arm before joining the children to watch cartoons. This choice protected Jack from being exposed to her self-harm.	
2. Sam was sitting on the lounge in a dissociative state. She was caught up in painful memories and feelings from the past. Her daughter Mia wanted to play, and came over to Sam, tapping on her leg and calling to her “Mummy, Mummy.” Sam felt irritated by this, and conflicted about providing for her young daughter when she didn’t receive nurturance in her own childhood. Sam had a choice to yell at Mia, and push her away, or to put aside her worries and memories for a little while to be with Mia and play with her in the present moment. She chose to join in the play session, which strengthened her bond with Mia and allowed her to experience positive new feelings to replace older ones.	
3. Sam was having a fight with her boyfriend over the phone and was becoming increasingly distressed. The children were in the kitchen, and Ethan was packing lunches and getting everyone ready for school. Sam was feeling abandoned by her boyfriend, and had a choice to seek further support from Ethan and ask him to stay home from school to be with her, or to take charge of getting the children to school before seeking support from appropriate services, so that she would not burdening her son. Choosing to ensure all children attended school helped them maintain contact with their learning and peers, and provided respite so that Sam could attend counselling.	

### Evaluation of the program - clinician acceptability

Research has identified the need for clinicians to develop greater understanding, training, and skills in working with mothers with mental illness [24] and individuals with personality disorder [25, 26]. Interactive training focused on furthering knowledge, skills and reflective practice has been identified to foster hope and confidence in clinicians when working with individuals with BPD [27, 28]. Thus, training in the brief intervention model involved a one-day interactive skills-based workshop to ground attendees with the theory, research, and implementation of the intervention manual and approach. The training was delivered by a team of PhD level clinical psychologists with specialised training and supervised clinical experience in the treatment of personality disorders. The training provided an opportunity for clinicians to reflect more broadly on their own clinical work with personality disorders through case studies and discussion of clinical problems.

This study aimed to investigate whether clinician engagement with this brief intervention model was acceptable. It is therefore an initial step in the evaluation of this model, which in future will include controlled trials with patients. We were interested whether clinicians rated this approach as helpful for improving treatment outcomes, and useful in changing their attitudes, knowledge and clinical skills in working with people with personality disorder, across clinician levels of expertise. We further aimed to explore what specific modes or aspects of learning the clinicians found most helpful in the brief intervention model. We hypothesised that providing clinicians with a brief intervention model targeting knowledge, skills, reflection and resources for working on parenting, an identified area of difficulty for people with personality disorder, would result in positive attitude change for clinicians in working with this client group.

## Method

### Participants

A total of 170 Mental Health and Drug and Alcohol staff attended one of the six single-day Project Air Strategy Parenting with Personality Disorder training workshops conducted during a one month period across four sites. Attendance at the six days of training ranged from 20–36 people per workshop. Institutional Review Board approval was received and all participants were informed of the aims of the study. A total of 168 clinicians provided informed consent. The staff were asked to provide professional details, recorded in the results section below.

### Data collection

Following training, clinicians completed a survey where they were asked to rate their satisfaction and perceived helpfulness of the brief intervention model for improving outcomes, whether they would recommend the brief intervention model, and the usefulness of the brief intervention model in making positive changes to their attitudes and skills with regard to working with people who have personality disorders. The items used were based on a previous study of attitudinal change following training for personality disorders [27].

Several other ratings were also obtained from participants following training. First they were asked to rate their self-reported level of expertise in treating personality disorders as minimal, developing, sound or advanced. Second, they were asked how satisfied they were with the brief intervention training program, and how helpful they felt it was for improving outcomes for people with personality disorders on a four point likert scale (not satisfied to very satisfied, and not helpful to very helpful, respectively). Third, on a four point likert scale (not useful to very useful) participants were asked how useful they found the brief intervention model for improving attitudes towards working with people who have personality disorder - specifically, their willingness, optimism, enthusiasm and confidence. Scores on the four attitude items were summed and a mean score derived. Participants were also asked to provide detailed comment in writing on the factors that they felt were important for promoting engagement with the brief intervention content and resources. These written transcripts were used in the qualitative analysis as described below.

### Data analysis

Descriptive data is presented and the general linear model was used to determine whether satisfaction, perceived helpfulness, recommendation of training and attitudes and skills varied according to profession and self-rated expertise. Qualitative data was thematically analysed using QSR Nvivo Version 10 [29]. The themes identified in the Nvivo analysis were subsequently analysed using Leximancer [30]. In this study, Nvivo was used to identify the main factors (Nvivo themes) that participants felt were important for promoting engagement with the brief intervention content and resources. Leximancer was then used to further explore and understand how the identified factors and the key words were used within the text.

Relationships between Nvivo themes were analysed by manually seeding the key words within the Nvivo themes as Leximancer concepts. A similar methodology was employed in a recent study exploring the attitudes of health professionals towards people with personality disorders [25]. It is important to note, a ‘concept’ in Leximancer does not represent a single word, but rather a constellation of related thesaurus words. By manually seeding the concepts of interest, we were also able to generate a theoretically meaningful thesaurus. For example, words such as ‘tools’, ‘fact-sheets’, ‘manual’ were all suitably grouped within the concept of ‘supported resources’. This process was done collaboratively with all members of the research team, and based on actual text coded within the Nvivo themes. The use of Leximancer in textual analysis is well supported in health and medical research [25, 31, 32], and has demonstrated satisfactory face validity, stability, and reliability [33].

## Results

### Participants

Attending clinicians were predominantly female (*n* = 145, 86.3 %), with an average age of 41.17 years (*SD* = 11.33, range 23–67). For the 168 clinicians who gave consent to participate, staff professions included nurses (*n* = 58, 34.5 %), psychologists (*n* = 51, 30.4 %), social workers (*n* = 32, 19.0 %), and occupational therapists (*n* = 13, 7.7 %). ‘Other’ professions included Aboriginal mental health clinicians, speech pathologists, and diversional therapists (*n*-14, 8.3 %). Most clinicians rated their level of expertise with working with people who have personality disorders as ‘developing’ (38.6 %) or sound (32.5 %), and just over one quarter rated their expertise as advanced (25.3 %). Only 3.6 % rated their expertise as minimal.

### Evaluation of the brief intervention model

**Fig. 1:**
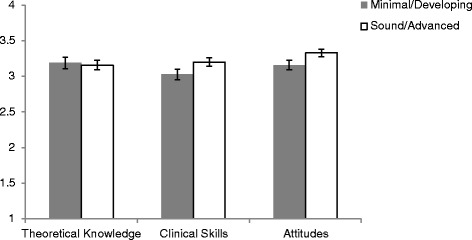
Mean Clinician Ratings of Acceptability of the Brief Intervention. Legend: The scale scores are from 1 indicating ‘not useful’ to 4 indicating ‘very useful’

Ninety-four percent (94 %) of responding clinicians (*n* = 168) reported being either satisfied or very satisfied with the brief intervention model training, and 95 % reported that they felt the brief intervention model would be helpful or very helpful in improving outcomes for parents with personality disorder. All clinicians (100 %) also reported that they would recommend the brief intervention model to a colleague. Ratings of usefulness suggested that overall, 83.6 % of clinicians felt the brief intervention model to be useful or very useful in improving their general attitudes towards people with personality disorder. Similarly, 88.1 and 85.7 % indicated the brief intervention model was useful or very useful in improving their theoretical knowledge and skills, respectively. Figure 1 provides the mean ratings for theoretical knowledge, clinical skills and attitudes for those with minimal or developing self-rated expertise as well as those with sound or advanced self-rated expertise (*N* = 168). Ratings did not differ significantly according to self-rated expertise, or professional group.

### Clinician reported helpful modes of learning

All but three responding clinicians provided written responses for qualitative analysis, meaning 165 independent responses were analysed using Nvivo and Leximancer. The Nvivo analysis identified four main themes within clinician responses that align well with the initial goals of this program (1) structured resources to support clinicians, (2) reflections and take home messages, (3) improved theoretical knowledge and clinical skills, and (4) dynamic and interactive training. A description of the themes and comments that comprise them are described below. The proportion of responses accounted for by each theme is in parentheses, following each title. Some responses spanned across a number of the themes. In these cases, responses were coded to multiple themes where appropriate. Hence, when totalled, the proportion of responses accounted for by each theme exceeds 100 %.

#### Use structured resources (47 %)

Many of the comments within this theme focused on the resources provided in the brief intervention, with reference to the DVD, help and fact sheets, and written resources (i.e. manual, intervention description). Clinicians reported the intervention was useful, practical and easy to implement in a clinical setting. Comments included reference to the usefulness of having filmed vignettes and written resources as tools that could be useful in the therapeutic setting to initiate difficult discussions about parenting, and could also be used to help parents empathise with their children. Clinicians felt the manual was clear, and the outline of the brief intervention model was useful, and easy to implement within a therapeutic setting.

#### Allow sufficient reflection on parenting challenges (28 %)

The brief intervention model and resources allowed clinicians to be reflective – on their own practice and on the issues arising for people with personality disorders that may not necessarily arise in therapy (i.e. parenting skills). Within this theme, four key take home messages were identified by clinicians: (i) ‘Recognition and validation’: Clinicians reported that their feelings that this client group can be *“inherently difficult”* to work with was recognised and validated, and felt more motivated and confident to tackle parenting discussions to improve outcomes for their clients. (ii) ‘Impact of personality disorder on the family unit’: Clinicians reported that the brief intervention model and resources helped them reflect on the issues that people with a personality disorder face when they are parents. Specifically, some clinicians indicated that it highlighted the need to reflect on and take the time to consider how the personality disorder can impact on children, and aspects of their social, mental and physical development. (iii) ‘Empathic approach’: Clinicians reported feeling more empathic to clients with personality disorder. Reflecting on the lives of a parent with a personality disorder, and viewing things through their perspective allowed clinicians to feel more empathetic and understanding of some of the difficult presentations. (iv) ‘Therapy doesn’t need to be complex’: The resources provided, particularly the manual and brief intervention emphasised that the core principles are basic including the importance of keeping clients in the *“here and now”* and initiating discussions on day to day parenting challenges is easy to undertake.

#### Ground training in improving theoretical knowledge and skills (26 %)

Providing specific parenting skills within a model focused on personality disorder, increased knowledge and clinical skills to work with this group. The demonstration and practice of skills and strategies (i.e. mentalisation, mindfulness) in the context of parenting were seen as avenues to engage in with clients who have a personality disorder.

#### Ensure sufficient time for interactive learning (17 %)

Clinicians benefited from a conversation style of the training that emphasised sharing experiences and engaging in clinical discussion. These interactive learning activities promoted confidence and reinforced specific skills for clinical practice.

### Leximancer analysis

**Fig. 2:**
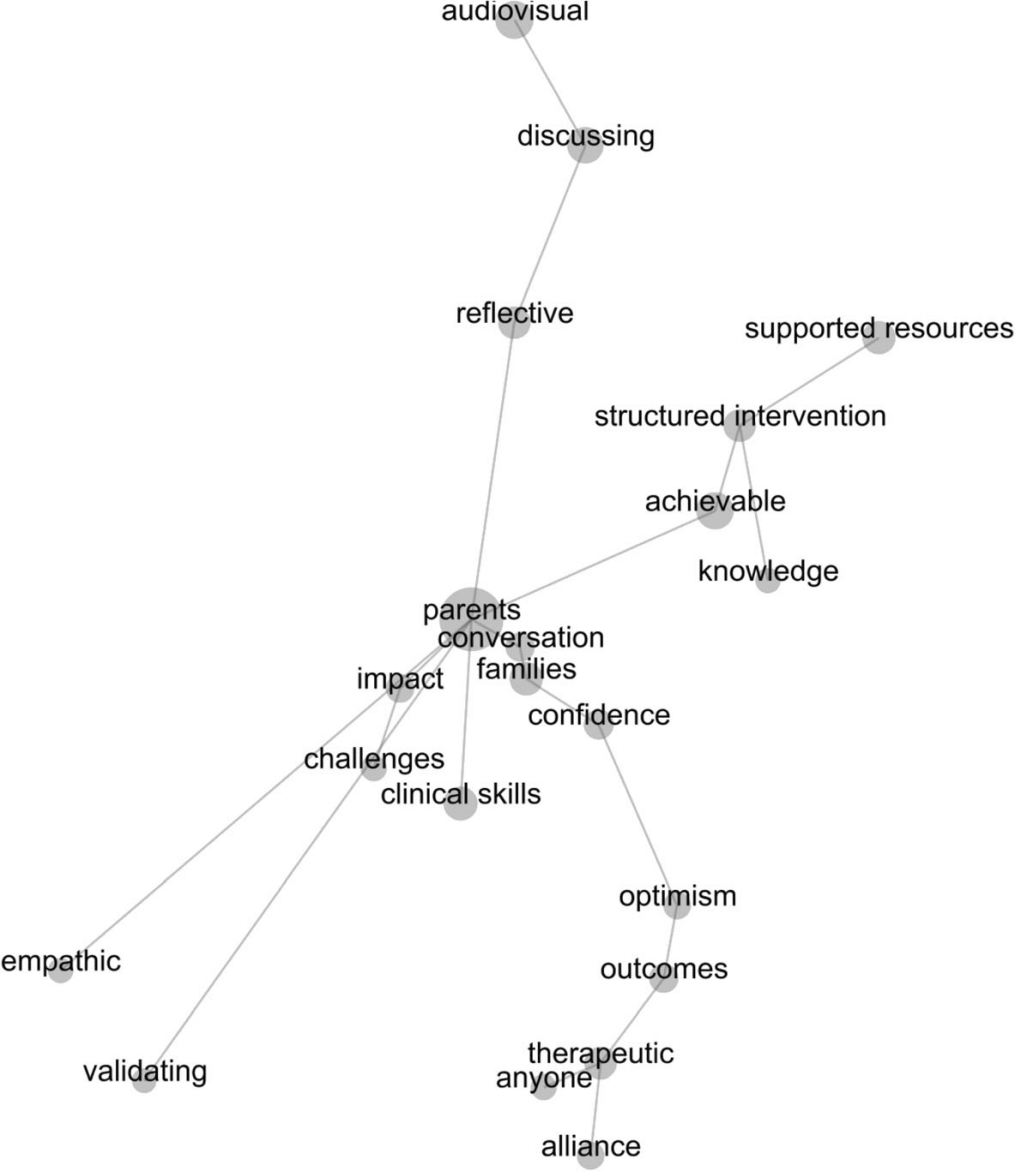
Leximancer concept map of clinician identified core features of the brief intervention model (*n* = 165)

A manually seeded Leximancer concept map (Fig. 2) was generated based on identified themes and selected key words in the Nvivo thematic analysis. The concept map (Fig. 2) shows the relationship between the main factors identified by participants as being important for promoting engagement with the brief intervention content and resources. Interpretation of the concept map was made with consideration of several factors: (i) the size of the dots– with larger dots indicating greater occurrence; (ii) the distance between the concepts – reflects how closely the concepts were used together in the text; (iii) familiarity with the text – understanding and familiarity in which the concepts were used in the raw data [30]. Interpretation was made in collaboration with the full research team.

The most salient concept in the text was ‘parents’ with 100 % of all other identified concepts linked to it directly or indirectly through other concepts. Thesaurus words for ‘parents’ included variations of the terms personality disorder, client, consumer, patient, and parents – hence, in this instance the concept of ‘parents’ represents people with personality disorder who are parents. Off this central concept extend three main arms, which contain groups of several other concepts, and a constellation of several other independent concepts (i.e. empathic, validation, clinical skills) that are semantically linked to ‘parents’ but to few other concepts. The three main arms represent groups of concepts that are strongly linked and close in proximity within the text. For instance, the concept ‘audiovisual’ is most frequently linked to words such as ‘discussing’ and ‘reflective’. With consideration of the raw text, this represents clinicians comments around the importance of audiovisual case study resources for promoting discussions (with peers and clients), and allowing them to reflect on their own practice and client perspectives.

Another arm of concepts with close semantic proximity to one another in the text include ‘supported resources’, ‘structured intervention’, ‘achievable’ and ‘knowledge’. This combination of concepts summarises the clinician’s comments that a structured intervention is achievable with the knowledge and information necessary to incorporate these ideas and use them to support their own practice with clients who have a personality disorder. The concept of ‘achievable’ was strongly linked with ‘supported resources’ and ‘structured intervention’, but upon closer investigation of each concept also occurred less frequently but with close semantic proximity to other concepts such as ‘optimism’, ‘outcomes’, and ‘confidence’ that are more directly related to one another in a third arm. This third arm is comprised of the concepts ‘conversation’, ‘families’, ‘confidence’, ‘optimism’, ‘outcomes’, ‘therapeutic’, ‘anyone’ and ‘alliance’, representing clinicians comments that therapeutic alliance is important, and that they feel more confident in initiating conversations with clients (parents) about their ‘families’.

The concepts ‘empathic’ and ‘validating’ are only linked directly to ‘parents’. Given the concept of ‘parents’ includes thesaurus words such as patients, clients, and consumers and based on raw text, this would indicate that clinicians feel empathic towards ‘parents’ with a personality disorders, and they feel validated that they can work effectively with this client population. The ‘clinical skills’ concept also extends independently off ‘parents’ and is most frequently used with this concept, however it does also occur with close proximity in the text with ‘families’, ‘challenges’, ‘therapeutic’, ‘structured intervention’, and ‘achievable’. The smaller connected arm comprised of ‘impact’ and ‘challenges’ – representing the recognition of the challenges that parents with personality disorder face and the impact it has on their lives and families.

## Discussion

This study describes clinician acceptability of a new parenting skills approach to working with people with BPD who are parents. Clinicians provided feedback in regards to the perceived helpfulness of the model, and the impact of the training on clinician attitudes and skills. The interactive workshops enhanced knowledge of personality disorder and the key struggles that parents with the disorder face. The brief intervention model was well received by clinicians, with the vast majority reporting that they were satisfied, and that they perceived that the approach would assist in improving client outcomes. After completing training in the model, clinicians reported the training was useful in increasing their willingness, optimism, enthusiasm, confidence, theoretical knowledge and clinical skills in working on parenting skills of those with personality disorder. Expert clinicians benefited as much as those who had less skills in personality disorder treatment. Training activities that emphasised case study analysis, reflective learning activities, and skills based intervention practices were well received. Semantic analysis revealed that clinicians thought that structured intervention and resources including audiovisual case study material promoted reflection, discussion, and knowledge regarding engaging in difficult conversations around parenting capacity and child protection.

The findings reported here resonate with previous studies that found clinician training in brief interactive workshops led to improvement in attitudes, knowledge and skills [27, 34]. Considering the literature indicating that stigma is commonly experienced in the community and within mental health services by parents with mental illness and particularly parents with personality disorder [24, 35, 36], and further, that non-pejorative clinician attitudes towards clients with BPD have been linked to improved client outcomes [37], the perceived usefulness of this training on clinician attitudes found here is an important outcome of this brief intervention model, and one that hopefully translates into the practice of the clinicians, although further research is required.

Importantly, this study took a further step in asking clinicians about their perspective on the core features of the brief intervention model, in order to formulate some suggestions for engaging clinicians in workshops on parenting with personality disorders, which may have positive impacts on clinician work with parents with personality disorder. Qualitative analysis of clinician responses revealed many themes indicating what they found most helpful in the brief intervention model. These can be summarised as ensuring there are ample case studies to engage reflection, ensuring sufficient opportunities for group discussion to promote sensitive practice, and grounding the training in skills based activities using clinical resources. Providing clinicians with tools to integrate into their practice as soon as they completed the training workshop increased their confidence, with clinicians reporting that they now had targeted strategies to implement with clients.

There were also some limitations to this study. Whilst the self-report questionnaire asked clinicians about how much they felt their attitudes had improved due the training, this study was limited in that data was not available for clinician attitudes prior to engaging with the brief intervention model, and there was no control group. Future research would be beneficial to follow-up clinicians to see if attitude change was retained over time, and also importantly, to measure outcomes for the parents and families who engage in the brief intervention.

## Conclusion

Supporting and building protective family factors and reducing family stress and risk factors is important in the treatment of mental health issues for parents, the protection of children and in the prevention of the intergenerational cycle of mental illness. The brief intervention model evaluated in this study can be readily added to current evidence-based treatments, with clinicians across differing levels of expertise perceiving it to be helpful and useful in improving their own attitudes, knowledge and clinical skills for working with parents with personality disorder.

## Abbreviations

BPD: Borderline personality disorder
